# Integrating Diverse Points of View on Intelligence: A 6P Framework and Its Implications

**DOI:** 10.3390/jintelligence9030033

**Published:** 2021-06-23

**Authors:** Robert J. Sternberg, Sareh Karami

**Affiliations:** 1 Department of Human Development, Cornell University, Ithaca, NY 14853, USA; 2 Educational Psychology Faculty, Mississippi State University, Starkville, MS 39762, USA; skarami@colled.msstate.edu

**Keywords:** 6P’s, adaptation, intelligence, process, person, press, problem, product, purpose

## Abstract

This article introduces a 6P framework for understanding intelligence, as well as the theories and tests that are derived from it. The 6Ps in the framework are purpose, press, problems, persons, processes, and products underlying intelligence. Each of the 6Ps is considered in turn. We argue that although the purpose of intelligence is culturally universal, the other Ps can vary at least somewhat over time and space. A single theory or test of intelligence represents a particular configuration of the 6Ps, but other configurations of the 6Ps might yield different theories and different tests.

Diverse theories of intelligence present intelligence in very different terms. Our goal in this article is to discuss the roles that purpose, press, problems, persons, processes, and products underlying intelligence currently play in theories and tests of intelligence, and the roles they ideally should play. Our consideration in this article is of explicit theories of intelligence proposed by intelligence theorists, although many of the same issues could be applied as well to implicit (folk) theories.

Some theories pinpoint internal origins of intelligence, others, external origins, and still others, interactions between the two. We discuss each kind of theory in turn.

Several kinds of theories stress internal origins. For example, psychometric theories represent intelligence in terms of factors (e.g., Carroll 1993; Cattell 1971; Johnson and Bouchard 2005; McGrew 2009; see De Boeck et al. 2020; Kaufman et al. 2020, for discussions of such theories). Similarly, cognitive theories offer sets of information-processing components (Hunt et al. 1975; Sternberg 1985) or of cognitive systems, such as of working memory (Conway and Kovacs 2013, 2020; Engle and Kane 2004; Kyllonen and Christal 1990), or of systemic characteristics—such as speed of information processing (Deary and Stough 1996; Jensen 1998; Nettelbeck and Lally 1976). Finally, biological theories are also offered as identifying the bases of intelligence in the brain. For example, P-FIT theory (Haier 2020a, 2020b; Jung and Haier 2007) is a general theory of intelligence suggesting that intelligence is largely or wholly biological. Intelligence is viewed as distributed in the brain. The theory is focused particularly upon the integration of functions in the frontal and parietal lobes of the brain. Barbey and his colleagues (Barbey et al. 2013; Barbey et al. 2012) have offered a cognitive architecture stressing the importance of dorsolateral prefrontal contributions to intelligence.

At the same time, there are other theories of intelligence that suggest that intelligence is more externally derived, depending on the cultural context in which people live (see Rogoff 2003; Sternberg 2020b). For example, Berry (1974) has offered a view of intelligence as radically culturally relative, whereby each culture invents its own conception of what intelligence is and then, essentially, acts as though whatever it believes about intelligence is what intelligence is. The Laboratory of Comparative Human Cognition (1982) took a more moderated view, arguing that culture affects what intelligence is but that the nature of intelligence is more conditioned on culture than absolutely determined by culture. Greenfield (2020), moreover, has suggested that intelligence can differ not only with respect to culture, but also with respect to secular time—that as cultures evolve, the nature of intelligence within that culture changes. Thus, at a given time, intelligence may be different things in different cultures because those cultures have evolved differently.

Systems theories of intelligence have taken a somewhat moderated stance (Sternberg 2020e). Sternberg (2004) suggested that the mental processes of intelligence are the same across cultures, but that the ways in which these processes are manifested in the environment are not universal and actually can be quite different. On this view, intelligence has both internal and external origins. The so-called analytical/componential part of the theory, which specifies information processes, would apply cross-culturally, but the creative and practical parts, which specify what constitutes creative or practical contributions in a societal context, can differ greatly from one culture to the next. Gardner’s (2011) theory of multiple intelligences is more like factor theories in specifying a fixed set of, in Gardner’s case, multiple intelligences that are common across cultures. However, the weights or relative importance of each of those multiple intelligences might differ from one culture to another. For example, musical intelligence might be important in one society and of absolutely no importance in another. Therefore, systems theories allow for both similarities and differences in the nature of intelligence across time and space.

There are various basic views of intelligence. Two of them are fundamentally different from each other in a key respect. Sternberg (2004) summarized the difference as follows. One view is that intelligence is an inherently biological, trait-like construct that is universal (referred to as Models I and II in (Sternberg 2004)). On this view, intelligence can manifest itself in different ways as a function of place or time, but it always results from the same basic biological substrate. The other view (referred to as Models III and IV in (Sternberg 2004)) is that intelligence and its manifestations can differ somewhat from one sociocultural manifestation to another—that what is adaptive and hence what constitutes intelligence as a measurable construct, actually can be different across space and time. The framework expressed here views intelligence always as adaptive, but what is adaptive can vary over place and time. Hence, the framework comes closer to the latter than the former view. 

We suggest, however, that there is not a “correct” answer to which of these views is correct. Rather, they represent somewhat different metaphors of mind (Sternberg 1990). Metaphors of mind are non-disconfirmable. They are ways of seeing the world and the phenomena within it. Hence, it is unlikely that contextualists will convince adherents of a narrower psychometric view that they should broaden their conception of intelligence, any more than the psychometrists are likely to convince contextualists that they should narrow their viewpoint. There is room for both viewpoints in the intelligence literature, because they address the phenomenon of intelligence in fundamentally different ways, each of which can teach us something about the phenomenon.

## 1. A 6P Framework for an Integrative Understanding of Intelligence

Rhodes (1961) proposed a 4P model that is sometimes applied to the understanding of creativity (Rhodes 1961). According to Google Scholar, as of 1 December 2020, the article has been cited 2339 times (https://scholar.google.com/scholar?q=Rhodes,+M.+(1961).+An+analysis+of+creativity.+The+Phi+Delta+Kappan,+42:+305-310.andhl=enandas_sdt=0andas_vis=1andoi=scholart, accessed on 18 June2021), so it has had substantial heuristic impact on the field of creativity. The 4P analysis was not presented as a “theory” of creativity, but rather as a useful framework for understanding different aspects of creativity.

We propose here an expansion of Rhodes’s (1961) model and apply it instead to intelligence. This is not a theory of intelligence or even a model of intelligence. Rather, it is a framework for understanding different aspects of intelligence and their interrelations. The framework suggests that, often, differences among theories of intelligence result not in those theories being mutually incompatible, but rather in their dealing with different aspects of intelligence. The six Ps in regard to intelligence will refer to the (1) Purpose of intelligence, (2) environmental/situational Press that produces intelligence as manifested in behavior—or, to put it another way, the Place in which intelligence is utilized, (3) the nature of Problems requiring intelligence, (4) cognitive and metacognitive aspects of Persons who are intelligent, (5) psychological Processes underlying intelligence, and (6) Products of intelligence. The two Ps we add to Rhodes’s framework are Purpose and Problems, for reasons we hope become clear in the article.

We hasten to add that there is nothing etched in stone or uniquely “correct” about the 6P framework we use. Others might prefer different frameworks for understanding intelligence. Moreover, one could argue that all of the elements of the framework having “P” as their initial letter might have resulted in compromises in the selection of the elements, originally by Rhodes (1961) and then by us. However, we note that each of the elements also has synonyms that begin with letters other than “P.” One instead could have referred to a *process* as an “operation” (the term used by Guilford (1967)); *person* as easily could have been “individual,” which historically, of course, has been the unit of analysis in differential psychology (Chamorro-Premuzic et al. 2015); *press* could be “context” (Sternberg 2021); *problem* could be “task”; *product* could be “solution,” and *purpose* could be “goal.” Our point is that the use of 6Ps did not truly constrain our choice of elements in the 6P model. Rather, the constraints came from the range of functions of intelligence needed for a framework for understanding the phenomenon of intelligence and theories of it.

We hope that the article will show that the framework is useful, as might be other frameworks, for understanding how particular configurations of the 6Ps can yield certain views on intelligence, whereas other configurations (for example, different environmental press) might yield different views or, for that matter, tests of intelligence. In other words, theories and tests of intelligence implicitly represent choices of Ps within which to frame intelligence, choices that the theoreticians and test-developers may not even be aware they made. Disagreements may result from different choices, but the choice often comes to be viewed as necessarily inhering in nature rather than in the researchers’ choices among the 6 Ps. 

If, indeed, intelligence has different aspects as revealed differentially by different Ps in the framework, one might wish to reconsider some of the assumptions that underlie the use of the terms “intelligence” and “tests of intelligence.” Employing only some of the Ps, we suggest, leads to an incomplete theory.

Why do we need a 6P framework for intelligence is the first place? Our suggestion is that the 6P framework encourages a theorist of intelligence to consider, in their proposed theory, aspects of intelligence that they otherwise might have neglected to consider but should consider. As an example, a typical psychometric theory might address aspects of the person (alleged sources of individual differences) but may fail to consider other Ps, such as process, press, problem, product, or purpose. Should a comprehensive theory of intelligence deal with these other issues? Would any legitimate theorist of intelligence care about what is missing? Apparently so. 

The 1970s and 1980s saw a rebellion of sorts against the dominance of psychometric theories by some cognitive psychologists, who suggested that a comprehensive theory of intelligence needed to highlight cognitive *processes*, not just hypothetical sources of individual differences across *persons* (e.g., Hunt et al. 1973; Hunt et al. 1975; Pellegrino and Glaser 1980; Sternberg 1985). Soon afterward, a group of theorists of intelligence argued that all these theories were inadequate because they failed to take into account environmental *press*, or context (e.g., Berry 1974; Laboratory of Comparative Human Cognition 1982; Mpofu 2004; Scribner 1984; Serpell 1974; Sternberg 2004). Some intelligence theorists have argued that both the *problems* and the *products* of conventional psychometric intelligence testing are inadequate, trivializing intelligence as it exists in the real world (e.g., Ceci 1996; Gardner 2011; Sternberg 2020e). Additionally, other theorists have argued, from the start, that the *purpose* of intelligence is adaptation to the environment (Binet and Simon 1916; Wechsler 1940) and that somehow, this purpose has gotten lost in our theories and assessments of intelligence (Sternberg 2020c, 2021). Although some approaches emphasize reasoning and problem-solving skills essential to adaptation to the environment (Lakin and Kell 2020), much of the work has been with abstract problems that are quite removed from the demands of everyday life

Not every theory or test of intelligence needs to be complete with respect to the 6Ps, but what the 6P framework does do is to point out how a given theory is either more or less comprehensive in the aspects of intelligence it deals with. The problem in the “intelligence business” has been not so much the incompleteness of theories and tests, but rather, failure of theorists and test-constructors to recognize their incompleteness. Oddly, the psychometrician who best recognized this problem was J. P. Guilford (1967, 1982), whose theory receives little attention today. Guilford attempted systematically to cover his bases by using a faceted model that took into account content, operations, and products. Although by the standards of the 6P framework, his theory still would be considered to be incomplete, it probably was more comprehensive than anything that came before it, and more comprehensive (although flawed) than most theories that have come after it. However, can any given theory or test of intelligence, no matter how comprehensive, even apply with any degree of universality?

First, one might pause at the notion that the same intelligence test can apply anywhere, essentially at any time (what is called “Model I” in (Sternberg 2004)). The idea is that, to make a test work cross-culturally, one need only translate it and make certain adaptations, such as to the vocabulary words or perhaps to general-information questions. However, the problem is that the language of the test and of the items in a test may not tap equivalent constructs across cultural groups. The environmental *press* is different in different places. In other words, test *problems* may not have the same meaning across cultural groups, resulting in different *persons* showing up as intelligent in one given setting versus another. The problems all measure something; the question is whether they measure the same thing, given that the environmental press vary from place to place and what is socialized as adaptive may also differ from one place to another.

Second, if intelligence is indeed fully universal and fully understandable through a single theory, then with careful planning, one can, at least in theory, construct a test that is culture attenuated. Attempts at cultural attenuation have been made with the *Army Beta*—a test used during World War I to screen applicants who needed a nonverbal test (Yerkes 1921)—the *Raven Progressive Matrices* (Raven et al. 1992), and the so-called *Cattell Culture-Fair Test of g* (Cattell and Cattell 1973). However, studies have shown that none of these tests has been particularly culture attenuated and certainly not culture free (e.g., Lee et al. 2020; Lewis et al. 2007; Lohman and Gambrell 2012). This would make sense in the 6P framework, again, because environmental *press* differs from one place to another, so that the *problems* that best measure intelligence also may differ. 

Third, if intelligence is truly IQ, and IQ can be measured anywhere, it becomes possible to believe that IQ is valid and that it is relatively straightforward to compare cultural groups, socially defined races, or even nations on their intelligence (e.g., Herrnstein and Murray 1994; Lynn and Vanhanen 2002; Rindermann 2018). If intelligence is IQ, and IQ is essentially the same thing for one group and another, why not just compare groups on IQ? It would be seen as wholly valid. However, if the way to measure IQ might value across environmental *press*, requiring different *problems* yielding possibly different *products* and thus *persons* identified as intelligent, then one might pause at the notion that mere comparisons of IQs across groups or nations are fully meaningful.

Now let us consider the elements of the 6P framework individually.

## 2. Purpose

Although definitions of intelligence vary and conceptions of how to study it vary as well, there seems to be good agreement among students of intelligence regarding its purpose, namely, adaptation. In an early symposium (Thorndike 1921), several contributors, such as S. S. Colvin, L. L. Thurstone, and R. Pintner, specifically mentioned, in one way or another, adaptation to the environment as the purpose of intelligence. In a later symposium on the nature of intelligence (Sternberg and Detterman 1986), adaptation to the environment again came out as a major point of consensus regarding the purpose of intelligence. Gottfredson (1997), representing 52 signatories to a statement on intelligence published in the *Wall Street Journal*, referred to intelligence as making sense of things and then figuring out what to do, which would seem like adaptation to the environment in other words.

Of course, there is more to intelligence than just adaptation to the environment. In the 1921 symposium, for example, many other characteristics of intelligence are mentioned, such as ability to think abstractly (L. M. Terman), the capacity to acquire capacity (H. Woodrow), judgment and reasoning (N. E. Haggerty), and the ability to learn (W. F. Dearborn). However, these various skills all could be viewed as serving the common purpose of adaptation to the environment. For example, we learn and reason well in order to further our adaptation to environmental demands and opportunities.

Adaptation to the environment can be seen narrowly or broadly (Sternberg 2019, 2021). The narrow form is when one changes one’s behavior better to fit the environment one is in. However, a broader form, as the term is used here, also involves shaping environments to make the environments a better fit to oneself, and selecting new environments, where possible, when the environment one is in just cannot be made to fit one’s needs or desires and when one is unable or unwilling to shape the environment to be a better fit.

Biologically, adaptation to the environment is the sine qua non for intelligence. When Charles Darwin spoke of natural selection, he spoke of it in terms of fit of organisms to environments. Intelligence is what enables organisms to improve that fit in order to ensure the survival of themselves and their future gene pool. Of what use is intelligence if one receives high scores on intelligence tests but then proceeds to act in a fashion that harms one’s own successful adaptation or, at a broader level, the success of the adaptation of future generations, including from one’s own gene pool? In practice, many educators and psychologists seem to have implicitly accepted Boring’s (1923) statement that intelligence is whatever intelligence tests test, without thinking through the implications, for example, that the notion is extremely conservative because it means that, whatever the tests happen to measure in a given time and place, that is labeled as intelligence. Boring had expected notions of intelligence to expand over time, which they have, but the measurement of intelligence as IQ and related constructs has continued unabated. The view of intelligence as what intelligence tests measure is also circular because the tests are supposed to measure a construct of intelligence, which in turn is defined in terms of whatever the tests happen to measure. 

Sternberg (2019, 2021) has proposed a notion of “adaptive intelligence” that is based on the notion that the primary purpose of intelligence is adaptation to the environment. The basic idea is that adaptation is defined in this theory not just as the usual individual criteria of success—grades in school, amount of schooling, health, marital success, success in career, and so forth—but also in terms of criteria that are relevant to humanity. That is, if someone has done very well in terms of individual criteria, but then has engaged in actions that potentially impair life for future generations, such as contributing purposefully to global climate change by, say, burning down Amazonian rain forest for profit, one would have to question whether they are adaptively intelligent. Adaptive intelligence applies not only at the individual level but also at the collective level (see also Malone and Woolley 2020), in that the decisions we make affect those all around us. For example, if someone has a high IQ, but in the time of the COVID-19 pandemic, makes no effort to wear a mask or to socially distance, thereby putting not only their own life at risk, but also the lives of others, it is not clear what it means to refer to the person as “intelligent.” 

We would argue that the need for adaptation to the environment is culturally and temporally universal. What constitutes adaptation to the environment can be different from one place or time to another, but the need for adaptation never goes away, as Darwin realized. Humans are susceptible to the same evolutionary pressures they always have been susceptible to, and that other species are susceptible to as well. These pressures will never leave, no matter when or where one lives. Therefore, we view the purpose of intelligence as universal.

Why does this matter? It means that intelligence is important everywhere. Some would argue that only individual outcomes matter—such as income, awards, grades, merit badges, or whatever. However, these are not biological bases for natural selection but, rather, socioculturally derived, extrinsically motivated measures of merit. For example, grades in school, income, occupational prestige, job-performance ratings—the values of outcomes such as these are socioculturally determined. Health outcomes are biological, but it is not adaptive when people prolong their own lives at others’ expense, as is happening worldwide where resources are being hoarded in some cases by the ultra-well-off at the expense of those who can barely eke together a living. For example, in some countries, including the United States, the wealthy have access to excellent healthcare while the poor often do not. The resources are not shared. The poor are prevented from being able to adapt the environment by their being poor. However, then, predictably, some scientists will point to the correlation of IQ or some other cognitive measure with health, perhaps failing to point out that those who are poor do not have the access to healthcare, in those countries, that would enable them to be healthy. In sum, adaptation to the environment is fundamental to intelligence, wherever one lives, but not everyone has equal access to the resources that would enable them to adapt and thus manifest their adaptive intelligence. 

For some, the term “’purpose’ may seem problematic.” From the perspective of this article, the perceived problematicity of purpose is itself a problem. Without a clear purpose for intelligence, the field risks being theoretically vacuous. Suppose someone asked a memory psychologist the purpose of “memory” and was told, instead of that the purpose of “memory” is “to encode, store, and retrieve information,” or just to “remember” things, rather that the question was itself problematical. What would one conclude? That evolution designed a complex mechanism like memory that has no adaptive purpose? 

We believe that the question of purpose for intelligence needs to be taken seriously and that some of the problems that have beset the field arose precisely because the field did not take this question seriously. Is the purpose of intelligence to account for adaptation, as Binet, Wechsler, and many early theorists (Thorndike 1921) suggested? Or is it only to predict culturally defined criteria of success, where the ability to achieve success on those criteria may itself be dependent on society’s perception of one’s intelligence? Is the purpose of intelligence to provide factorial coherence? Or what? If we avoid these questions, as theorists of intelligence, we do so at our own peril.

## 3. Press

Press refers to those environmental pressures and peculiarities that stimulate people to use their intelligence in particular environments. Press are extremely important to the understanding of intelligence, because they illustrate how different the demands are on adaptation from one time or place to another.

For example, in studies in rural Kenya, Sternberg and colleagues (Sternberg et al. 2001) found that a crucially important element of adaptation to the environment was knowledge of how to recognize the symptoms of various parasitic illnesses and then of how to treat the various illnesses with natural herbal medicines. Children in these environments did not, in general, have access to Western anti-parasitic medicines—they did not know of them and their parents would have been unable to afford them if the parents had known of them. Instead, they treated the illnesses with herbs that had been found, over the course of time, to be effective. Treating the illnesses was adaptively important because the effects of the illnesses on their lives were substantial—loss of time at school or at work, inability to concentrate, serious physical symptoms, and in extreme cases, death (such as from malaria or extreme cases of other diseases such as trichuriasis). The researchers created a test that was effective in distinguishing different levels of practical, actionable knowledge and skills in treating the parasitic illnesses.

An unexpected finding was that scores on the test of practical knowledge and skills correlated negatively with tests of fluid and crystallized abilities. There was a simple reason for this: In rural Kenya, the children who were perceived as “intelligent” were removed from school early because they could be societally useful in other ways. The children who were not perceived as “intelligent” were left in school. Hence, oddly, the children who were most valued by the local society received less formal education and acquired less academic knowledge and fewer such skills but at the same time acquired more practical knowledge that they could use to protect their health—possibly their lives—as well as the health of other children and adults.

In 2001, when the article was published, skills in preventing, recognizing, and treating illnesses, as best as was possible, may have seemed peripheral to intelligence—conventional intelligence tests measured and still measure instead skills like solving number series problems or knowing the meaning of the word “assuage.” However, today, it is worth reexamining how the kind of environmental press that made knowledge and skills about treating illnesses in rural Kenya is relevant the world-over. 

As we write (late-2020), a pandemic of COVID-19 is sweeping the world. Although there still is undoubtedly value in solving abstract problems, certainly there is special value now in knowing and acting on the kinds of knowledge the Kenyan children needed—how to prevent, recognize, and treat (outside hospitalization) an illness that is serious and possibly fatal to many people. Those who act unintelligently, in the sense of intelligence as adaptation, risk not only getting sick and dying, but infecting untold numbers of others through their lack of adaptive intelligence. The number of infected cases has increased from 2,850 in January 2020 to 16,519,940 in July 2020 (STAT News 2020). It only took a few months for the skill set that constitutes intelligence as adaptation to change. In particular, skills needed to avoid contracting COVID-19 became more and more important.

One might argue that the behavior changed but that the underlying skills did not. However, the negative correlation between the measures of adaptive intelligence and general intelligence in Kenya would argue for a different interpretation. They would argue that correlations between different tests are not some intrinsic fact of nature but rather determined in part by environmental press. Had the skills that were valued in rural Kenya been different, the pattern of correlations would have been different. Industrialized societies are more used to the most intelligent students staying in school longer. The schooling increases their academic knowledge and skills, resulting in higher IQ (Ceci 1996). We are used to industrialized societies where socialization of children results in correlations of tests between many kinds of adaptive skills being positive. Because, in these societies, more academic skills are valued in terms of moving children through the successively narrow funnel of the educational and then the occupational systems, socialization patterns produce positive correlations. As noted above, IQ should correlate with future health, if only the higher IQ individuals make the salaries that enable them to obtain quality health care, or in some cases, any health care at all. The press of the environment thus not only determines what is intelligent in a given time or place, but what it even means to be intelligent in an adaptive sense.

A second example is the set of adaptive skills needed to live in extreme environments, such as in the far North. The skills that, for example, Yup’ik indigenous people, especially children, need to cope with their harsh environments are different from those needed to cope in mainstream America or other industrialized settings, and these skills do not correlate well with general intelligence (Grigorenko et al. 2004). Again, the skills required to cope with harsh environments might seem far afield from the skills most people need to cope, or at least they did in 2004, when the article was published. However, today, with global climate change rapidly advancing, people all around the world are finding themselves forced to cope with harsh environments caused by climate changes in ways that would not have been readily predicted in 2004, or even, somewhat later. These harsh environments may actually develop important intellectual skills in children (Ellis et al. 2020), but not necessarily the ones tested by standard tests of general intelligence.

Consider another example of effects of environmental press on what it means to be intelligent, driving while black (DWB) (LaFraniere and Lehren 2015; Sides 2018). Data from 20 million traffic stops show that black people are twice as likely to be pulled over by police officers, even though they proportionately drive less than do whites. We also know they are more likely to be subject to police violence. Black men are 2.5 times more likely to be killed than white men during their lifetimes (Peeples 2020). As a result, black families teach black youth, especially males, skills they need in order to cope with the fact of life that they are more likely to be stopped and killed by police. One could argue, of course, that the police are merely doing what they perceive their job to be. However, to the black men who are stopped and possibly injured or killed, learning how to cope with an encounter with the police is an important survival skill in the United States, much as is dealing with parasitic illnesses in Kenya or, today, dealing with COVID-19 anywhere. 

Some scholars might say that the skills measured on intelligence tests are those that are specifically transferable to practically any specific concrete life situation. However, are they? Sternberg (2020a; see also Sternberg et al. 2000) as well many others (Irvine and Berry 1988) have argued and presented data arguing that the skills needed to cope with practical problems differ in multiple key respects from the skills needed to succeed on tests of general intelligence and that the transfer is relatively weak. 

Human intelligence can be and has been defined, at least in part, in terms of transfer of training (Ferguson 1954). However, transfer of intellectual skills is extremely elusive and difficult to obtain (Blume et al. 2010; Detterman and Sternberg 1993; Gick and Holyoak 1983). The only way to get transfer is to teach for transfer, and this often is not done (Detterman and Sternberg 1993). Abstract-reasoning skills are useful, but whether they are transferable to specific domains is open to question. The environmental press may just be too different. 

In conclusion, sources of environmental press are, at least in part, temporally and spatially particular, not universal. These sources of press affect how intelligence manifests itself in every society. The skills needed to adapt to different environments are often different. It may well be that general intelligence is of some use, no matter what the environmental press. However, there is more to intelligence than general intelligence and having a Mensa-level IQ may help in some societies, especially in gaining admittance to high-IQ societies, but may be more or less central, depending on closely the press of the environment match what general-intelligence tests measure.

## 4. Problems

As a result of different kinds of environmental press, the kinds of problems that constitute intelligent thinking can be very different under different circumstances, as alluded to above. For example, a major adaptive problem for rural Kenyan children, as discussed above, is understanding and treating parasitic diseases, so a problem Sternberg et al. (2001) used to measure adaptive-intellectual skills was:

- “A small child in your family has homa. She has a sore throat, headache, and fever. She has been sick for 3 days. Which of the following five Yadh nyaluo (Luo herbal medicines) can treat homa?
  - Chamama. Take the leaf and fito (sniff medicine up the nose to sneeze out illness).*
  - Kaladali. Take the leaves, drink, and fito.*
  - Obuo. Take the leaves and fito.*
  - Ogaka. Take the roots, pound, and drink.
  - Ahundo. Take the leaves and fito.’’

Problems such as this require a blend of different kinds of knowledge and skills. These kinds of knowledge and skills generally are not learned in school but rather in everyday life. They require the test-taker to know what homa is. However, children do not sit down, as a medical student might, and memorize treatments for homa. Rather, they learn by observation and by inferences from those observations regarding what works and what does not.

Today, an adaptive-intellectual problem in much of the world might consist of when and where to take steps such as wearing a mask, social distancing, or washing hands as efforts to avoid contacting COVID-19. Although these steps might sound basic, huge numbers of people are not following them. The knowledge is of little use if not put into action, which is why the problems in adaptive-intelligence research need to be put in terms of procedural, not just declarative knowledge.

Should there be a separate “P” for “problems,” as opposed, perhaps, to sticking with the original Rhodes (1961) 4 Ps. We believe problems need carefully to be considered because the characteristics of many problems in the real world (Sternberg 2020d) are different from those offered on intelligence tests. Adaptive-intellectual problems differ from many of those found on general-intelligence tests in a number of important ways. In particular, the problems differ in terms of the:

- *Type of answer required.* Conventional intelligence tests and their proxies, such as the ACT and SAT (Frey and Detterman 2004; Koenig et al. 2008; Sackett et al. 2020), generally require multiple-choice or short-answer responses. Problems of adaptation to the everyday world general require responses that are extended. For example, there is no multiple-choice solution as to how to stop the COVID-19 pandemic.
- *Structure of the problems.* Intelligence-test problems tend to be well-structured, with one path or sometimes more than one clear paths to solution. Adaptive problems tend to have no clear path to solution, as with the problem of COVID-19.
- *Level of emotional arousal induced.* Intelligence-test problems tend to be emotionally sterile. Real-world problems, such as that of COVID-19, tend to be emotionally arousing.
- *Contextualization in terms of everyday life demands.* Adaptive problems in everyday life are highly contextualized to the demands of particular environments. Intelligence-test problems tend to be purposely very weakly contextualized so that they will be understandable by a variety of test-takers.
- *Stakes for adaptation in everyday life.* The stakes of individual intelligence-test problems are low, even if the results of tests, combined across all problems, are higher. In contrast, in real-world adaptive problems, such as that of COVID-19, the stakes of a single problem are high; bad decisions can result in serious reverses in one’s life circumstances, illness, and death.
- *Need for recognition of the existence of problems.* Intelligence-test problems stare one in the face. Recognizing they exist is trivial. Real-world problems often are hard to recognize. COVID-19 has spread so widely around the world because, by the time leaders recognized it was a problem or their jurisdictions and not just a region of China, it was too late. Recognizing that traditional conceptions and tests of intelligence potentially can be problematical also has been challenging for many in the field.
- *Need for the definition of the problems.* Intelligence-tests generally define problems for the test-takers. They are clear in what they are asking and in what is required to answer. Real-world problems are often ill-defined. It is not exactly clear what the problem is. For example, with COVID-19, even expert medical advice on how to avoid it has varied, as scientists have tried more and more to understand exactly what symptoms the disease causes and why it causes those symptoms.
- *Time allowed for solution.* IQ-type test problems tend to be answerable in short periods of time. Real-world problems, in contrast, tend to need to be solved over long stretches of time, such as is the case with COVID-19.
- *Recurrence.* IQ test problems, once a test is handed in, are gone. One no longer needs to confront them. Real-word problems, in contrast, are hard to get rid of and often keep coming back, as has been the case with COVID-19.
- *Need to search for information.* In conventional IQ-test problems, the information one needs to solve the problem is in the problem. One needs to draw on prior knowledge and skills, which one may or may not have, but the problem makes clear what information is needed to solve the problem. In a real-world adaptive problem, the information is not readily available. Often, it is not clear where, if anywhere, one can find it.
- *Need to evaluate the validity of information.* One generally can presume that the information given in an IQ test is valid, at least for the solution of the problem. Test-constructors do not generally provide fake information. In the real world, one has to evaluate all the time whether information is real or fake. With COVID-19, there may well be more fake information than real information.
- *Knowledge needed to solve the problems.* Real-world problems require large amounts of tacit or informal knowledge to solve (Polanyi 1976). This knowledge is acquired from life experience. It is different from the formal knowledge measured by many standardized tests of intelligence and achievement.

These differences matter. Consider two examples.

First, in research on scientific reasoning, Sternberg and colleagues (Sternberg and Sternberg 2017; Sternberg et al. 2017; Sternberg et al. 2019) found that the demands of the environment in doing scientific research lead to the development of kinds of intellectual skills that differ at least somewhat from those measured by standardized tests. Clearly, both general intelligence and scientific reasoning require analytical-reasoning skills. However, the skills from general-intelligence tests appear not to transfer all that well. In the research, tests of scientific reasoning—formation of alternative scientific hypotheses, designing scientific experiments, and drawing scientific conclusions, and even analyzing scientific teaching—tend to correlate moderately with each other, at least among university students at a selective university. However, they do not consistently correlate with tests of general intelligence, and some of the correlations were negative. Therefore, the transfer, or at least, positive transfer appears to be modest.

One might argue that such skills should not necessarily correlate with scores on tests of intelligence, as the tests of intelligence do not measure these skills, but then the question is why STEM graduate programs, all of which *do* require these skills, rely heavily on tests that measure skills largely different from rather than similar to the skills most directly required for occupational success in STEM professions. Thus, even kinds of reasoning or problem solving that are needed to satisfy the press of an occupation may be largely different from those required by general-intelligence tests, even though they would appear on the surface to be at least moderately positively related. 

The level of transfer could be increased, Sternberg et al. (2019) discovered, by making the scientific-reasoning problems multiple-choice rather than open-ended. The problem is that real-life scientific reasoning is virtually never multiple-choice. No one presents a scientific researcher with a multiple-choice decision regarding choices of experimental designs and asks the researcher to pick the best one. Rather, the experimenter has to figure out the experimental design for him or herself. 

Second, in research on practical intelligence (Hedlund 2020; Sternberg et al. 2000), researchers have found that scores on a variety of tests of practical knowledge and skills—practical intelligence—predict real-world job performance about as well as do IQ tests but that the scores on these tests correlate weakly, if at all, with the IQ tests. The practical tests provide situational-judgment tests (SJTs), where respondents have to indicate how they would solve the real-world, job-related problems presented. Intelligence tests just are not good predictors.

To summarize, problem content on tests of intelligence is probably not universal, although some other kinds of content probably are universal, such as are needed to make an intimate relationship succeed or to raise children. Additionally, cultural and temporal particularity matter. The people who are good at solving certain types of problems are not necessarily good at solving other types of problems. In the past it was believed that the solution to this problem was culture-free or culture-fair intelligence tests, as noted earlier. However, this solution proved to be extremely problematical. Such tests, usually of abstract reasoning, proved to be more susceptible to the Flynn effect than were verbal tests of crystallized abilities (Flynn 1987, 2012, 2016). In other words, the supposedly culturally reduced tests were more susceptible to cultural influences than were the tests that were supposed to be more culturally loaded (Brouwers et al. 2009). If one wants to study intelligence as adaptive, then one needs to present problems that are realistic with respective to the adaptive demands that individuals (and groups) will confront.

## 5. Persons

What are the characteristics of persons who are intelligent, and are they universal? Psychometric theories, such as John Carroll’s (1993) theory and McGrew’s (2009) CHC (Cattell–Horn–Carroll) variant of it, specify a rather long list of abilities that are alleged somehow to lie within the head. In Carroll’s “three-stratum” theory, there are three levels. Two of the levels—the top and the bottom—are identical to Charles Spearman’s (1927) theory, with general intelligence (*g*) at the highest level of the hierarchy (Stratum III), and fairly specific abilities at the lowest level of the hierarchy (Stratum I). The level that distinguishes Carroll’s theory is the middle one (Stratum II), with fluid intelligence, crystallized intelligence, general memory and learning, broad visual perception, broad auditory perception, broad retrieval ability, broad cognitive speediness, and processing speed. Johnson and Bouchard’s (2005) theory, mentioned earlier, separates verbal, perceptual, and image-rotation abilities, rather than fluid and crystallized intelligence. Additionally, Howard Gardner’s (2011) theory is broader yet, including linguistic, logical-mathematical, spatial, musical, bodily kinesthetic, naturalist, interpersonal, and intrapersonal intelligences, which Gardner views as systems rather than as discrete abilities. The broadest model of all has been Guilford’s (1967, 1982; Guilford and Hoepfner 1971) Structure-of-Intellect (SOI) model, which at various times has consisted of different numbers of allegedly orthogonal abilities, generally ranging from 120 to 180. These abilities were divided into three dimensions of a cube: contents, operations, and products. This model has been questioned, having been based on a form of factorial rotation (Procrustean rotation) that later was shown to be of at least debatable value (Horn and Knapp 1973).

Other theories of intelligence as a property of the person have taken different forms. For example, recent cognitive theories have especially emphasized the importance of a person’s working memory (e.g., Conway and Kovacs 2013; Engle and Kane 2004; Kyllonen and Christal 1990), including the capacity of that working memory (Daneman and Carpenter 1980). Recent biological theories have moved away from Gardner’s view of intelligence as modular to a view of intelligence as fairly widely distributed in the brain, for example, as representing an interaction of the frontal, temporal, and parietal lobes (Jung and Haier 2007). 

As posed, there is a common meta-theory behind each of these diverse theories—that intelligence somehow resides in the brain and is waiting for situations in the world (environmental press) to elicit its use. Thus, intelligence, in these theories, is something to be discovered. This view is in contrast to the more radical cultural views, such as the view of Berry (1974), which is that intelligence is essentially invented anew by each culture. 

We suggest that the abilities themselves likely are universal. The fact that they are abilities, or latent sources of individual differences, does not imply that they are equally relevant, or indeed, at all relevant to intelligence as it exists around the world (Sternberg 2004). For example, broad visual perception may have much less relevance, if any, to a blind person than to a sighted one, and broad auditory perception may have much less relevance, if any, to a person who is deaf. 

The criterion we suggest for determining whether an ability is relevant to intelligence in a given time or place is necessity—is it necessary for adaptation to the environment (Sternberg 2019, 2020a)? For physically impaired persons, certain physically derived abilities are less important to intelligence. If one draws on Howard Gardner’s (2011) theory of multiple intelligences, bodily kinesthetic intelligence clearly was not necessary for the adaptation of Helen Keller or Stephen Hawking to their environment. In Sternberg’s (2020a) theory of successful intelligence, recognition of strengths and the ability to capitalize on them, and recognition of weaknesses and the ability to correct or compensate for them, are essential to (successful) intelligence. Keller, Hawking, and many others worked out methods of compensating for their physical debilities. 

The limitations on universality of particular abilities need not be confined to physical abilities. For example, in some religious cultures, music is, or at least certain kinds of music are, forbidden. In these cultures, musical abilities would count much less as an intelligence, if indeed they constitute an intelligence, than in other cultures. In preliterate societies, the verbal abilities needed for reading and writing certainly accounted for less than they do today in literature societies. In the past, the abilities needed to hunt, to gather, and to defend oneself against physical attack certainly would have been more important, and in lawless situations, those abilities remain important. These abilities still draw on conventional abilities, such as working memory capacity, but also go far beyond those abilities. That is, good working memory in itself will not turn one into a great hunter.

The abilities of the person qua abilities seem to be universal, but the abilities that constitute intelligence as an adaptive entity seem to be much more limited to particular times and places. As the demands of the world change, so do the abilities that together constitute intelligence as adaptation to the environment. The COVID-19 pandemic has highlighted such changes. The simple ability to protect oneself and others from disease has become much more important the world over.

## 6. Processes

Scholars using the cognitive approach to intelligence set out to provide an understanding of the mental processes underlying the factors that psychometricians had identified as comprising what they believed intelligence to be. 

The simplest approach to cognitive analysis was suggested by a number of scholars, including Arthur Jensen (1998), who proposed that choice reaction time was implicitly a measure of speed of neuronal conduction, and that the basis of intelligence is speed of such conduction. Nettelbeck and Lally (1976) proposed a related approach using a measure called inspection time, which is concerned with the amount of time it takes an individual to decide accurately which of two lines is longer. 

The earliest and perhaps seminal work on this paradigm was done by Hunt et al. (1973) and then Hunt et al. (1975). They introduced a “cognitive-correlates” approach to understanding intelligence. The idea was that the basic processes cognitive psychologists already were studying in their laboratories formed the basis for intelligence. As an example, Hunt and his colleagues suggested that speed of name retrieval for letter names, calculated by the subtraction method from a task used by Michael Posner (Posner and Mitchell 1967), might form a basis for verbal ability. As people age, their speed of retrieval generally increases (Hertzog 2019), which, according to these theories, would mean that the fluid aspect of their verbal intelligence as measured by retrieval speed declines. Detterman (1994) believed that if one could find the basic underlying information-processing components of intelligence, one eventually would be able to understand much of what constitutes general intelligence.

Sternberg (1985) introduced an alternative approach, which he called componential analysis, and which later came to be called “cognitive-components analysis” (Pellegrino and Glaser 1980). Sternberg suggested that there are three kinds of information-processing components of intelligence, which he views as universal: metacomponents, or executive processes; performance components, which execute the commands of the metacomponents; and knowledge-acquisition components, which learn how to do things in the first place. An example of a metacomponent is defining the nature of a problem; an example of a performance component is inferring the relation between two concepts. An example of knowledge-acquisition component is selective encoding, whereby one figures out what information available in a problem is relevant for solving it, and what information is irrelevant.

In the 21st century, the emphasis in information-processing research has switched to the capacity of working memory (as mentioned earlier) but also to the processes underlying it (as relevant here—Conway and Kovacs 2020; Ellingsen and Engle 2020; Engle and Kane 2004). Other investigators look into processes involved in complex reasoning (Lakin and Kell 2020), problem solving (Hambrick et al. 2020), and decision making (Gigerenzer 2020).

The accounts of processes refer to processes that we suggest vary in their universality. The metacomponents, or executive processes, are universal, because one needs them in order to adapt to any environment. One always has to define problems, for example, or allocate resources to their solution. As part of adaptation, one also needs working- and long-term memory to store and retrieve results of the operations of the various components. However, other processes are not universal. For example, although information always has to be retrieved from long-term memory, the kind of information may differ across cultures. It is not clear that the characters of Mandarin Chinese are retrieved in the same way as letters of the modern-day English alphabet. Additionally, in a preliterate society, there are no letters to be retrieved, even if individuals communicate orally. Therefore, we offer that metacomponents and knowledge-acquisition components are universal, but that performance components, those used to execute particular tasks, vary across time and space as a function of the particular tasks that are needed or viewed as adaptive in particular environmental contexts.

## 7. Products

The products of intelligence as adaptation clearly vary widely across time and space. Today, few people reading this article will need to learn to operate farming implements—manual, electric-powered, or gas-powered. Yet, there are many people in the world who live off subsistence farming and there were even more, proportionate to population, in the past. If you need to go to the city to work and there is no public transportation, you may need to learn to operate a bicycle or an automobile, but if you do not, you may not need to operate either one. 

Today, producing a well-cited scholarly article or book may be viewed as a great achievement for a professor, but if you work for the CIA, writing a book and not getting sufficient clearance may land you in prison. In a dictatorship, that book may get you killed. Some products of intelligence are valued virtually anywhere—solutions to surviving a threat to one’s life, such as the decision to fight back or to run away, as advisable—whereas other products are valued only in limited settings—for example, answers to IQ tests for those applying for admission to Mensa or another high-IQ organization. Therefore, products, like processes, vary from culturally universal (fight or flight) to culturally specific (answering multiple-choice problems on standardized tests).

## 8. Conclusions

We have suggested that it may be useful to adopt a 6P framework in seeking to understand intelligence but also to understand the theories that have been proposed to characterize intelligence. On this view, different theories often look at either different P’s, or different aspects of the same Ps. Pitting the theories against one another may create misleading comparisons, because the different theories deal with distinctly different aspects of intelligence.

The problems people confront that are relevant to intelligence vary greatly across time and place. The problems faced by people in very cold climates, for example, are quite different from the problems faced by people in very hot climates. The problems also change over time. More and more, people will have to use their intelligence to adapt to hot climates. Additionally, who would have expected, a year before we wrote this article, that they would have to use their intelligence to preserve their own health and that of others from COVID-19.

The characteristics of persons vary in their universality, with some, such as working-memory capacity, probably relevant anywhere, whereas others, such as auditory acuity, may vary. Regions of the brain are probably universal in some respects—parietal-frontal integration—but more specific regions of the brain may not only differ across time and place, but also across persons, because people solve the same problems in different ways (Sternberg 1985). People with lesions in the brain often devise methods of compensation so that they can solve problems using parts of the brain that others might not use (Gazzaniga et al. 2018). 

With regard to processes, metacomponents (executive processes) and knowledge-acquisition components are universal, but performance components (used directly to perform tasks) are not. That is, people will always need to do things such as recognize when they have a problem, but the processes they use to solve the actual problem will depend on the time and place in which they are confronting problems. For most people, the processes used to solve hunting animals for food are less relevant than they would have been some years back. In the future, they may become more relevant again, and there are still parts of the world where they are relevant today.

Finally, the products of intelligence differ widely across time and space. Products that might seem quite ordinary to many of us today—such as answers to multiple-choice tests—may seem totally bizarre to people in other times and places. 

Although we have built our analysis on a 6P framework, which is in turn an extension of a 4P framework, there are other frameworks that could have been used as well. For example, a related theoretical framework, proposed by Glaveanu (2013), is a 5A framework. The 5As in this model are actor, action, artifact, audience, and affordances. The *actor* is the creative person or, if there is more than one, persons. The actor is analogous to the person in the 6P framework. The *action* is essentially the process in the 6P framework. The *artifact* is analogous to what is called the product in the 6P framework; but it additionally encompasses the sociocultural context in which the product is created. Glaveanu specifies two different and distinct elements for press. The *audience* is analogous to the social aspect of press in the 6P framework. Additionally, part of press in the 6P framework is what Glaveanu refers to as the *affordance*, which is a sort of material press. It involves the relationship between the actor and whatever surrounding objects are relevant in the material environment. We note that the 6P framework applies to other higher order constructs as well, such as wisdom (Sternberg and Karami 2021a) and creativity—where we have proposed 8Ps (Sternberg and Karami 2021b).

The results of this analysis suggest that some aspects of intelligence probably are universal and others are not. Hence, it is doubtful whether there is a single test (or number) that will measure all aspects of intelligence in a way that could be said to be universal with respect to place and time. In the future, one could imagine a detailed anthropological analysis of what constitutes adaptive behavior with different kinds of environmental press in different places. One might end up with tests, based on such analyses, that varied in the kind of problems they contain and the kinds of products they require. At present, that is an open question. However, the question is worth addressing before we simply assume that placement of a theory or test of intelligence in a particular configuration of the 6P’s is universal across time and space.
